# Health and care workers’ perceptions of PPE and physical distancing for COVID-19: A qualitative evidence synthesis

**DOI:** 10.4102/jphia.v16i2.621

**Published:** 2025-04-17

**Authors:** Dachi I. Arikpo, Afiong O. Oku, Okwu A. Onyema, Edward O. Odey, Hannah Hamilton-Hurwitz, João P. Toledo, Kathleen Dunn, April Baller, Helen J. Smith, Martin M. Meremikwu

**Affiliations:** 1 Cochrane Nigeria, Institute of Tropical Diseases Research and Prevention, University of Calabar Teaching Hospital, Calabar, Nigeria; 2 Department of Community Medicine, University of Calabar Teaching Hospital, Calabar, Nigeria; 3 Department of Sociology, University of Calabar, Calabar, Nigeria; 4 Department of Social Science Education, University of Calabar, Calabar, Nigeria; 5 World Health Organization, Geneva, Switzerland; 6 Public Health Agency of Canada, Ottawa, Canada; 7 International Health Consulting Services Ltd, Merseyside, United Kingdom; 8 Department of Pediatrics, University of Calabar Teaching Hospital, Calabar, Nigeria

**Keywords:** COVID-19, SARS-CoV-2, infection prevention and control, PPE, masks, physical distancing, physical barriers, qualitative studies, qualitative evidence synthesis, systematic review

## Abstract

**Background:**

Despite the effectiveness of personal protective equipment (PPE) and physical distancing interventions for COVID-19 infection prevention and control (IPC), low uptake among health and care workers persists.

**Aim:**

To synthesise evidence from primary qualitative research exploring the perceptions and experiences of health and care workers on the use of PPE and physical distancing in the context of COVID-19.

**Setting:**

Healthcare settings including care homes.

**Method:**

An electronic database search was conducted using search terms based on the inclusion criteria, and the search strategy was peer-reviewed by a team of information scientists. Thirty qualitative studies were sampled after eligibility screening independently by two review authors. Synthesis was performed using the thematic synthesis approach. The confidence in each review finding was assessed using the Grading of Recommendations, Assessment, Development, and Evaluations; Confidence in the Evidence from Reviews of Qualitative Research (GRADE-CERQual) approach.

**Results:**

Fifteen analytical themes were identified. Health and care workers valued the use of PPE in patient care. The sense of value was heightened by perceived susceptibility to infection with COVID-19, the need to deliver optimal patient care and the desire to protect family members. Service delivery, clinical workflows, the absence of visual cues for spatial separation and physical infrastructure hindered adherence to physical distancing guidelines.

**Conclusion:**

The gap between IPC guidelines and their implementation is an important health system barrier to PPE use and physical distancing in healthcare settings.

**Contribution:**

This review provides useful insights on key considerations for planning and implementing IPC in healthcare settings.

## Introduction

Infection prevention and control (IPC) strategies in the context of the COVID-19 pandemic (January 2020 – May 2023) aim to limit and control the spread of the severe acute respiratory syndrome coronavirus 2 (SARS-CoV-2) in the healthcare settings. As a part of the global COVID-19 public health emergency response, the World Health Organization (WHO) has emphasised the importance of the core components of IPC programmes, adhering to the hierarchy of control measures and the implementation of standard and transmission-based precautions.^1^ Physical barriers and distancing are key non-pharmacological interventions categorised within the hierarchy of controls framework for minimising exposure to infectious diseases such as COVID-19.^2^

Health and care workers face a disproportionately higher risk of COVID-19 because of occupational exposure and can contribute to the spread of hospital-acquired infections if appropriate IPC measures are not instituted and complied with.^3,4^ The WHO provides guidelines on measures to prevent and contain the spread of COVID-19. However, the availability of these guidelines alone is insufficient to ensure the implementation of IPC in the context of COVID-19; cases of non- or poor adherence to IPC guidelines for COVID-19 by health and care workers are common.^5,6,7^ Effective IPC in healthcare settings depends on health and care workers’ adherence to guideline recommendations.

Personal protective equipment, including gowns, gloves, face masks, respirators and eye protection work by providing a protective barrier from infectious particles and other body fluids during healthcare activities.^2^ Engineering controls work by reducing the risk of transmission of infections in healthcare settings through the structure and design of the health facility. These interventions may include ventilation, installing air purification systems, structural changes to workspaces, installing physical barriers, designation of areas for specific procedures and activities, providing dedicated entry and exit routes in settings to maintain and enforce physical distancing and communicating respiratory etiquette and hand hygiene.

Non-adherence to IPC guidelines may be the result of varying perceptions and experiences of these measures and the context and conditions in which they are implemented. Thus, understanding if health and care workers who are directly affected by these guideline recommendations value these measures and perceive them to be acceptable, equitable and implementable for IPC in the context of COVID-19 allows for public health entities to craft recommendations that support implementation and adherence. Insights provided by synthesis of qualitative research aid our understanding of complex phenomena, behaviours, experiences and the contexts in which they occur. This understanding is necessary to improve the uptake and transferability of guideline recommendations to a range of contexts, cultures and individuals; and identify avenues to improve adherence.

This qualitative evidence synthesis (QES) was commissioned by the WHO to fill the knowledge gap in the evidence base for the 2023 update of the guideline on IPC in the context of COVID-19.^1^ The WHO uses a robust methodology for guideline development, which includes using qualitative data to inform relevant domains of the Evidence-to-Decision framework. The database search performed at the time of conducting this study showed no existing QES on perceptions and experiences of health and care workers on the use of PPE and physical distancing interventions in healthcare settings in the context of COVID-19. In addition to this gap was the urgent need to document experiences, perceptions and challenges of implementing these IPC measures during the COVID-19 pandemic in order to inform future pandemic preparedness and response.

Consequently, this review aimed to (1) synthesise available qualitative research exploring the perceptions and experiences of health and care workers on the use of PPE and physical distancing interventions in healthcare settings in the context of COVID-19 and (2) identify the contexts and conditions that facilitate or hinder uptake and adherence to these interventions.

## Methods

### Design

This rapid QES was conducted using the Cochrane methodology,^8,9,10^ and reported using the ‘Enhancing Transparency in Reporting the Synthesis of Qualitative Research’ (ENTREQ) and Preferred Reporting Items for Systematic Reviews and Meta-Analyses (PRISMA) checklists.^11,12^ The protocol was registered and published in the International Prospective Register of Systematic Reviews (PROSPERO, CRD42022356383).

### Inclusion criteria

We used the SPICE (setting, perspective, phenomenon of interest, comparison and evaluation) framework,^13^ to articulate the inclusion criteria (Table 1). We included primary studies that used recognised qualitative methods of data collection and analysis; these could be standalone qualitative research or qualitative components of mixed-method studies. The literature search was targeted at primary studies published in response to the COVID-19 pandemic to inform the update of the WHO COVID-19 IPC guidelines. Only relevant studies published from 01 January 2020 to the date of the database search (05 September 2022) were eligible for inclusion.

**TABLE 1:** Inclusion criteria (SPICE framework).

Concept	Description
Setting	Healthcare facilities, including care homes Community including households in any geographical location and level of healthcare
Perspective (population)	Stakeholders: Health and care workers involved in patient care and those not involved in patient care Healthcare policymakers Health facility clients (including residents of care homes, recipients of care – inpatients, and outpatients) and visitors Community members – general public and members of households
Phenomenon of interest	Physical barriers and distancing interventions for COVID-19 infection prevention and control
Intervention	Physical barriers and distancing for infection prevention, including PPEs (e.g., face masks, coveralls, gowns, shoe covers, N95 respirators, gloves, goggles, face shields) Physical distancing (e.g., keeping a distance of a least 1 m or 2 m between patients or persons) Engineering controls (air cleaning and purifier technologies; spatial separation using physical barriers).
Evaluation (*outcomes*)	Perceptions of stakeholders, including views, attitudes, experiences, perspectives Factors influencing uptake (barriers and facilitators) at the individual, provider, health system, community, and social-political levels
Study design	Primary studies conducted using qualitative study designs, including ethnography, phenomenology, case studies, grounded theory studies, applied qualitative research, mixed-methods and process evaluations Studies using both qualitative methods for data collection (e.g., observations, focus group discussions, individual interviews) and qualitative methods for data analysis (e.g., thematic analysis, framework analysis, content analysis and grounded theory)
Date limits	01 January 2020 to 05 September 2022; to capture research published in response to the COVID-19 pandemic.

PPE, personal protective equipment.

### Search strategy

Three information scientists from Cochrane worked collaboratively to design and implement the peer-reviewed search strategy (Appendix 1). As this was a rapid review, one database – Ovid-MEDLINE – was searched. The reference lists of studies meeting the inclusion criteria were searched to identify any additional studies for inclusion.

### Study selection, sampling and data extraction

All search hits were imported into Endnote Reference Management software for eligibility screening and deduplicated.^14^ The eligibility criteria were applied to the remaining records. Authors worked in pairs, with one author (either E.O.O. or O.A.O.) screening all titles, abstracts, and full-texts of potentially eligible studies using a pre-piloted eligibility screening form. The second author (either D.I.A. and A.O.O.) in each pair verified all screening output.

Full-text screening yielded 78 studies for inclusion, too many to manage in a QES. Therefore, the maximum variation purposive sampling approach was used to select studies for inclusion in the synthesis.^15,16^ We developed a three-step sampling frame with the following parameters: ‘closeness of the study to our synthesis objective’, ‘geographical spread or representation’ and ‘data richness’. Data richness was determined using a data richness scale.^17^ Before applying the sampling criteria, we categorised each included study into intervention groups (PPE and masks in healthcare settings, masks in the community and physical distancing in healthcare and community settings).

A pre-piloted data extraction spreadsheet in Microsoft^®^ Excel was used to extract information on key study characteristics, interventions and findings reported by the sampled studies; separate spreadsheets were used to extract themes and supporting quotes relevant to the review objectives. One review author (O.A.O.) extracted data from the sampled studies and two authors (D.I.A. and A.O.O.) verified all extracted data for accuracy and completeness. All discrepancies in the study selection process and data extraction were corrected by verifying authors or by consulting a third review author (H.J.S.).

### Assessment of methodological limitations of sampled studies

Methodological limitations were assessed using an adapted version of the Critical Appraisal Skills Programme (CASP) tool for qualitative studies.^18^ The tool evaluated the appropriateness or adequacy of descriptions of the study context, sampling strategy, data collection and analysis, evidence supporting the findings, reflexivity and ethical considerations. Two authors (D.I.A., O.A.O.) appraised the studies independently and reached a consensus on the final judgement for each domain. We did not exclude any studies based on the quality assessment.

### Data analysis

For data relevant to health worker perceptions and experiences, two review authors (D.I.A. and A.O.O.) independently performed an initial coding of extracts to familiarise themselves with the data, identify discrepancies in coding and reach a consensus on the coding approach. Thereafter, one author (D.I.A.) performed line-by-line coding of extracted texts from the sampled studies and a second author (H.J.S.) verified the coding. The codes were identified, developed and reviewed iteratively based on the contents and meaning of the extracted texts.

Thematic Synthesis,^19^ allowed us to derive descriptive themes directly from the data and organise them using inductive and ‘constant comparison’ methods.^20^ Analytical themes were subsequently developed by re-grouping descriptive themes and generating analytical themes.^21,22^

Data on factors influencing uptake and adherence to IPC interventions were synthesised deductively using the Supporting the use of research evidence (SURE) framework.^23^ This framework lists factors that influence the implementation of a policy option at the level of the care recipient, care provider, health service and system constraints, and the social and political context. We extracted data against the supporting the use of research evidence (SURE) framework domains and, from these, developed descriptive and then analytical themes. The coding process and development of descriptive and analytical themes were discussed and agreed to by all authors.

### Assessing the confidence in the review findings

The GRADE-CERQual approach was used to assess the confidence level (high, moderate, low or very low) in each review finding (analytical theme).^24^ One review author (D.I.A.) assessed the confidence of each finding across the four domains. A second author (H.J.S.) verified the judgements and ratings. The overall assessment was based on the consensus of the author team.

### Review author reflexivity

As a review team, we understood that our perspectives and experiences of the COVID-19 pandemic may influence how we collect, analyse and interpret the data. Therefore, before the synthesis commenced, we discussed and reflected on our expectations about the phenomena of interest. This approach helped to minimise any bias in the synthesis process. All authors had experienced the COVID-19 pandemic and generally considered IPC strategies essential for mitigating the spread of the virus, but did not have clear expectations of the review findings. In addition, we believe that the number of review authors discussing and reaching a consensus on each review finding, the multidisciplinary nature of the author team and maintaining a reflexive stance throughout the conduct of this review helped provide rich insights, balanced views and ensured that we reached an accurate interpretation of the data.

### Ethical considerations

This is a systematic review of existing published qualitative research. This article followed all ethical standards for research without direct contact with human or animal subjects.

## Results

### Included studies

**FIGURE 1:**
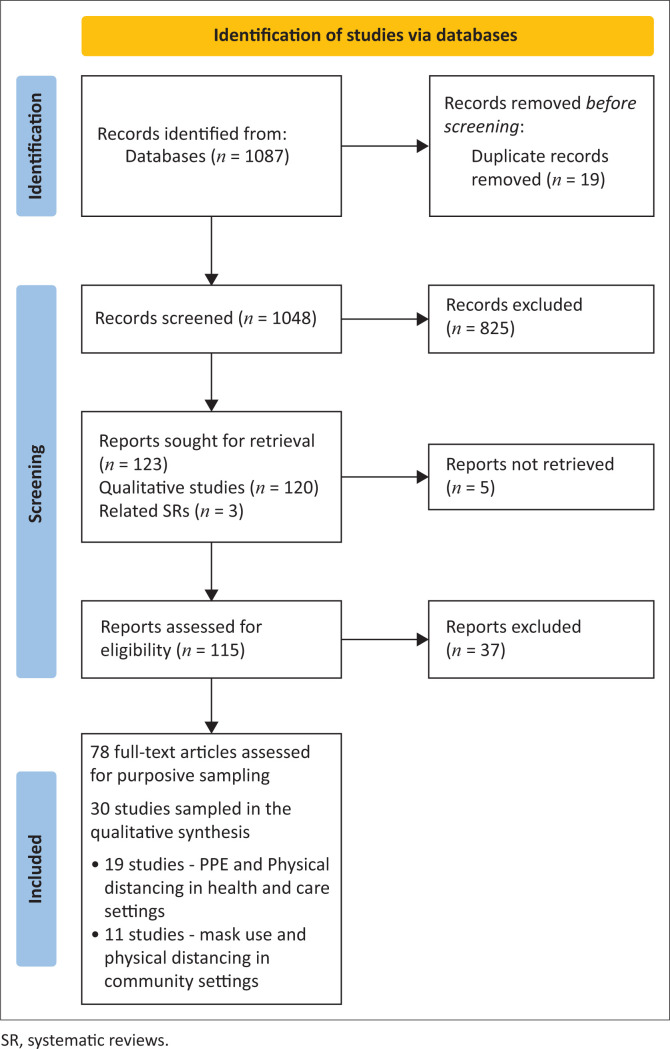
Preferred Reporting Items for Systematic Reviews and Meta-Analyses (PRISMA) flow diagram.

A total of 1067 records were identified in the electronic database search, including studies conducted in both healthcare and community settings. The records (titles, abstracts and full-texts) were subsequently screened for eligibility. At the end of the study selection process, 30 studies from both settings were sampled and synthesised in the QES (Figure 1). Post hoc, the author team agreed that it would be more useful to present the synthesis for each setting in separate reports. In this review, we present the results of the synthesis of 19 studies,^25,26,27,28,29,30,31,32,33,34,35,36,37,38,39,40,41,42,43^ on PPE use (*n* = 15) and physical distancing (*n* = 4) in healthcare settings. The synthesis of studies on mask use and physical distancing in community settings (*n* = 11) is reported elsewhere.^44^

Three of the sampled studies were set in two lower-middle-income countries (LMICs): India^27,28^ and Iran^30^ (Table 2). Four studies were from three upper-middle-income countries (UMICs): China,^26^ Indonesia^29,42^ and Turkiye.^35^ Twelve of the sampled studies^25,31,32,33,34,36,37,38,39,40,41,43^ were conducted in high-income countries (HICs): Australia,^25,40^ Finland,^31^ Italy,^33^ Portugal,^32^ Spain,^34^ United Kingdom (UK)^37,39^ and the United States (US).^36,38,43^ One of the studies^41^ was a multicountry study conducted across four HICs: Belgium, France, Italy and Luxembourg.

**TABLE 2:** Characteristics of included studies.

Study ID	Study setting	Aim	Study design	Perspective	Participants	Sample size	Data collection method	Context	Method of data analysis	Overall assessment of methodological limitations
**PPE or face masks (*n* = 15)**										
Sivaraman 2022^27^ LMIC (India)	Health facilities	To understand the behavioural intention to use PPE among the healthcare professionals working in otorhinolaryngology patient care areas	Primary qualitative study	Health and care workers	Otorhinolaryngology healthcare providers. Faculty (*n* = 2), junior or senior residents (*n* = 5), nursing officers (*n* = 3), and ward or OPD/OT attendants (*n* = 3) and audiologist (*n* = 2)	15	Interviews	Urban	Thematic analysis	Minor
Sharma 2022^28^ LMIC (India)	Health facilities	To explore barriers faced by HCWs while using PPE during a pandemic situation in a tertiary care hospital	Primary qualitative study	Health and care workers	Residents, nurses, and hospital attendants	21	Focus group discussions	Rural and urban	Framework analysis	Minor
Goodarzi 2022^30^ LMIC (Iran)	Health facilities	To explore the healthcare providers’ experiences of CPR in patients with COVID-19	Primary qualitative study	Health and care workers	Nurse (*n* = 16), nurse anaesthetist (*n* = 4), physician (*n* = 6),	26	Interviews	Urban	Thematic analysis	Minor
Setiawan 2021^29^ UMIC (Indonesia)	Health facilities	To explore strategies for overcoming challenges in caring for COVID-19 patients at hospitals in Indonesia based on health and care workers’ experience	Primary qualitative study	Health and care workers	Physicians (*n* = 12), nurses (*n* = 16)	28	Interviews	Urban	Thematic analysis – Collaizzi approach	Minor
Fan 2020^26^ UMIC (China)	Health facilities	To assess the difficulties faced by healthcare personnel in using PPE in clinical practice during COVID-19 outbreak in Wuhan, China	Primary qualitative study	Health and care workers	Healthcare personnel including nurses and doctors with experience working in surgical and medical wards during the COVID-19 pandemic	20	Focus group discussions	Urban	Thematic analysis	Moderate
Koken 2022^35^ UMIC (Turkey)	Health facilities	To explore the experiences of cardiovascular nurses working in a COVID-19 intensive care unit during the pandemic	Primary qualitative study	Health and care workers	Cardiovascular nurses	10	Interviews	Urban	Interpretive phenomenological analysis	Minor
Broom 2022^25^ HIC (Australia)	Health facilities	To document, through in-depth interviews, the experiences of HCWs in relation to infection prevention and control during the COVID-19 pandemic	Primary qualitative study	Health and care workers	Medical doctors (*n* = 20), nurses (*n* = 23), allied health (*n* = 9), non-clinical (*n* = 8), other (*n* = 3)	63	Interviews	Urban	Thematic analysis	Minor
Venesoja 2021^31^ HIC (Finland)	Health facilities	To describe frontline health and care workers and other authorities’ views and experiences on continuous use of surgical masks and respirators (face masks)	Mixed-methods study	Health and care workers	Emergency department personnel (*n* = 21), emergency medical services personnel (*n* = 82), other health professionals (*n* = 13), other authority (*n* = 4)	120	Online survey using open ended questionnaires	Urban	Content analysis	Minor
Ribeiro 2021^32^ HIC (Portugal)	Health facilities	To understand how therapists experience the therapeutic process using a facial mask	Mixed-methods study	Health and care workers	Psychotherapists with at least some experiencein face-to-face practice under the COVID-19 pandemic.	137	Online survey using open ended questionnaires	Urban	Thematic analysis	Minor
Ferrari 2021^33^ HIC (Italy)	Health facilities	To explore how PPE and social distancing have impacted nurses’ and children’s caregivers’ experience of communication and relationship during COVID-19 pandemic in an emergency and a general paediatric department	Primary qualitative study	Health and care workers	Nurses	17	Interviews	Urban	Content analysis	Minor
Romeu-Labayen 2022^34^ HIC (Spain)	Health facilities	To explore nurses’ experiences and perception of risk regarding the use of PPE during the first wave of the pandemic in Spain	Primary qualitative study	Health and care workers	Nurses from different healthcare settings (hospital, field hospital, home emergencies, primary care, nursing home)	29	Interviews	Urban	Thematic analysis	Minor
Hoernke 2021^37^ HIC (UK)	Health facilities	To report frontline HCWs experiences with PPE during the COVID-19 pandemic in the UK	Primary qualitative study	Health and care workers	Doctors (*n* = 28), nurses (*n* = 8), medical associate professional (*n* = 4), pharmacist (*n* = 2), other (*n* = 4)	46	Interviews	Urban	Framework analysis	Minor
Hayirli 2021^36^ HIC (US)	Health facilities	To categorise and describe barriers to teamwork posed by PPE and distancing in the emergency setting	Primary qualitative study	Health and care workers	Registered nurses (*n* = 18), physicians (*n* = 17), nurse practitioners and physician assistants (*n* = 7), pharmacists (*n* = 5), social workers (*n* = 3) medical assistants and technologists (*n* = 5)	55	Interviews	Urban	Thematic analysis	None
Clay 2022^39^ HIC (UK)	Health facilities	To understand the perspectives of pwCD around the impact of staff wearing face masks on the experience of stroke rehabilitation. To understand staff perspectives on how stroke rehabilitation for pwCD is affected by staff wearing face masks	Primary qualitative study	Recipients of care	People with communication difficulties post-stroke	11	Focus group discussions via Microsoft Teams	Urban	Reflexive thematic analysis	None
Markkanen 2021^38^ HIC (US)	Care homes	To characterise qualitatively the impact of the COVID-19 pandemic on three key HC stakeholders: clients, aides, and agency managers	Primary qualitative study	Health and care workers involved in patient care, home care clients	Directors and managers at home care agencies (*n* = 12); home care aides (*n* = 16); home care clients (*n* = 9)	37	Individual interviews	Urban	Grounded theory	Minor
**Physical distancing (*n* = 4)**										
Fauk 2022^42^ LMIC (Indonesia)	Health facilities	To investigate the challenges to adherence to COVID-19 preventive guidelines and practices	Primary qualitative study	Health and care workers	Medical doctors, nurses, and pharmacists	23	Interviews	Rural	Framework analysis	Minor
Stulz 2022^40^ HIC (Australia)	Health facilities	To explore midwives’ experiences about how COVID-19 impacted their ability to provide woman-centred care	Primary qualitative study	Health and care workers	Midwives	26	Interviews	Urban	Thematic analysis	None
Kane 2022^41^ HIC (Belgium, France, Italy and Luxembourg)	Health facilities	To examine how care professionals experienced this period and faced these new constraints weighing on their professional practices	Primary qualitative study	Health and care workers	Psychiatric professionals	96	Interviews	Urban	Thematic analysis	Minor
Keller 2022^43^ HIC (US)	Health facilities	To understand barriers to physical distancing among HCWs on an inpatient unit and identify strategies for improvement	Primary qualitative study	Health and care workers	Nurses, dietician, physical therapist, physician	20	Interviews and observations	Urban	Content analysis	Minor

Note: Please see the full reference list of the article Arikpo DI, Oku AO, Onyema OA, et al. Health and care workers’ perceptions of PPE and physical distancing for COVID-19: A qualitative evidence synthesis. J Public Health Africa. 2025;16(2), a621. https://doi.org/10.4102/jphia.v16i2.621, for more information.

ID, dentification; OPD, outpatient department; OT, operation theatre; HC, healthcare; LMIC, lower-middle-income countries; PPE, personal protective equipment; HCWs, health and care workers; CPR, cardiopulmonary resuscitation; COVID-19, coronavirus disease 2019; UMIC, upper-middle-income countries; HIC, high-income countries; UK, United Kingdom; US, United States; pwCD, people with communication difficulties.

Fifteen of the sampled studies^25,26,27,28,29,30,31,32,33,34,35,36,37,38,39^ reported perceptions and experiences of PPE in healthcare settings (Table 2). Four studies^40,41,42,43^ reported the perception of physical distancing interventions in healthcare settings. We did not find any studies on other forms of physical barriers. Seventeen studies were primary qualitative studies and two^31,32^ were mixed-method studies. Study participants included doctors, nurses, allied health workers, residents, otorhinolaryngologists, hospital attendants, nurse anaesthetists, pharmacists, medical assistants, medical technologists, directors or managers at home care agencies, home care aides, psychotherapists, emergency department personnel, emergency medical services personnel and other healthcare professionals.

The methodological quality of the studies ranged from no methodological limitation (*n* = 3 studies), minor methodological limitations (*n* = 15 studies) to moderate limitations (*n* = 1 study) (Table 2). Most studies provided descriptive information on the study context, sampling strategy, data collection and analysis approaches, and ethical considerations. They also provided underlying data to support their findings. Only eight studies^29,30,32,33,34,36,39,40^ explicitly reported on researcher reflexivity. The QES authors discussed the implications of these limitations on each finding.

### Qualitative synthesis findings

In all, 15 findings (analytical themes) were identified: 10 on PPE use, including masks and 5 on physical distancing (Table 3). The GRADE-CERQual assessment of the findings ranged from low to high confidence (Table 4).

**TABLE 3:** Synthesis results (themes and supporting quotes): Perceptions of personal protective equipment use and physical distancing in healthcare settings.

S/N	Analytical theme	Descriptive themes (review findings)	Studies contributing to the review finding	Supporting data (example quote)
1	Health and care workers value the use of masks and PPE in patient care	PPE gave peace of mind	Sivaraman, 2022^27^ (India)	‘When I began working with COVID patients, I was frightened of coming into contact with them. However, *knowing that my PPE covered my entire body provided me peace of mind* that I was not in direct contact.’ (Nursing Officer) (Sivaraman, 2022, India) [*italics added by author*]
		Masks and PPE create a safety climate to deliver optimal care	Sivaraman, 2022^27^ (India); Ribeiro, 2021^32^ (Portugal)	‘… [*T*]he face mask contributes to *creating a safety climate and protecting clients’ and therapists*’ physical health, making it possible to do face-to-face therapy.’ (Ribeiro, 2021, Portugal) [*italics added by author*]
2	Health and care workers were anxious about being put at risk	Anxiety and insecurity about the level of protection provided	Broom, 2022^25^ (Australia); Hoernke, 2021^37^ (UK)	‘I was looking at videos of my friends who were in other countries, because I have a lot of classmates from uni and we’re spread all over the world, and we were comparing PPEs and I was shocked with what we have. We were just having goggles, N95 mask, and also gown. Whereas with them, they are all covered with all those hazmat suits and with different layers as well and they’ve got all their hats and everything. Whereas I don’t even have any hat at all. So, for me, *I feel really, really insecure about the PPE that we had.*’ (ICU nurse Queensland) (Broom 2022, Australia) [*italics added by author*]
		Perception of susceptibility and risk	Hoernke, 2021^37^ (UK); Sivaraman, 2022^27^ (India)	‘The first thing to do is I … don’t make them feel like a pawn in a bigger game, because sometimes we feel like we are obliged to do stuff to save the rest, but we are part of the rest too.’ (Doctor, consultant) (Hoernke, 2021, UK) [*italics added by author*]
3	Masks and other PPE cause physical discomfort to health and care workers	Health workers experience physical discomfort wearing masks and PPE	Setiawan, 2021^29^ (Indonesia); Broom, 2022^25^ (Australia); Sivaraman, 2022^27^ (India); Hoernke, 2021^37^ (UK); Fan, 2020^26^ (China); Koken, 2022^35^ (Turkey); Venesoja, 2021^31^ (Finland); Markkanen, 2021^38^ (US); Ribeiro, 2021^32^ (Portugal)	‘During a long day at work, *wearing a mask can cause all sorts of trouble, such as headaches, a burning sensation in the lungs, the rubber bands abrading behind the ears, and eyeglasses becoming foggy*.’ (Venesoja, 2021, Finland) [*italics added by author*] ‘… And I think as well, wearing the N95 mask that *there’s an element of re-breathing, so it was quite tiring as well*. So, a 12-hour shift of caring for a patient with those precautions was quite tiring as well.’ (Broom, 2022, Australia) [*italics added by author*] ‘Of course, I must hold it if I want to take a pee, but if I want to defecate when I am still wearing PPE, … I am permitted by another member to leave …. (Setiawan, 2021, Indonesia)
4	Masks and other PPE challenge effective communication	PPE compromises communication with colleagues	Hoernke, 2021^37^ (UK); Hayirli, 2021^36^ (US)	‘I feel like it’s been a lot more challenging with the *PAPRs to hear each other very well.* […] *It’s hard to tell if people got you. I’ll say something* […] *but I don’t know that they heard me. I think that’s the hardest thing.* […] *So then, you’re saying three or four times instead of moving on*. Everything takes longer.’ (Hayirli, 2021, US) [*italics added by author*] ‘I think it does make you feel very… *dehumanised because you can’t recognise any of your colleagues*.’ (Hoernke, 2021, UK) [*italics added by author*]
		Masks and PPE affect communication and relationship with patients	Koken, 2022^35^ (Turkey); Ferrari, 2021^33^ (Italy); Setiawan, 2021^29^ (Indonesia); Sivaraman, 2022^27^ (India); Clay, 2022^39^ (UK); Markkanen, 2021^38^ (US); Ribeiro, 2021^32^ (Portugal)	‘When I talk, with mask and at a distance, they [*patients*] *would not fully understand what I am speaking, and this led to misunderstandings and arguments.*’ (Sivaraman, 2022, India) [*italics added by author*] ‘*The reduction in facial cues may result in other non-verbal cues being heightened, or nuance normally conveyed by facial expression being lost. The intended message can therefore be misinterpreted* … It’s a bit like a text message *… it can be perceived in lots of different ways … patients are not keeping up with who’s being spoken to … non-verbal cues that are really important to aid their understanding are either removed or dampened down because of the masks.*’ (Clay, 2022, UK) [*italics added by author*]
		Learning to communicate effectively with patients while wearing a mask	Ribeiro, 2021^32^ (Portugal); Ferrari, 2021^33^ (Italy)	‘… [*T*]herapists talked about reinforcing the *use of active listening and interviewing skills, encouraging the verbal expression of emotions, trusting in the therapeutic process,* and asking for supervision … Find some alternatives to *help the client signal what he is feeling,* be aware that you will need more time to understand the client.’ (Ribeiro, 2021, Portugal) [*italics added by author*]
5	Familiarly with masks and other PPE helped health workers adapt for COVID-19	Already familiar with PPE pre-pandemic	Sivaraman, 2022^27^ (India)	‘… [*S*]ince we were *already using surgical masks*, we became comfortable with its daily usage very easily.’ (Sivaraman, 2022, India) [*italics added by author*]
		Progressive adaptation over time occurs with masks	Ribeiro, 2021^32^ (Portugal)	‘Therapists’ indications that *early discomfort, feelings of strangeness, or difficulties were progressively solved and as well as mentions of a progressive adaptation to the use of the face mask* in the therapy context. In this regard, one therapist said: “It is uncomfortable in the beginning since we are unable to see the entire face and identify the facial expressions so well, *but over time this discomfort disappears*”.’ (Ribeiro, 2021, Portugal) [*italics added by author*]
6	Health and care workers in care homes experienced unequal access to PPE	Inequality in access to PPE	Romeu-Labaven, 2022^34^ (Spain); Markkanen, 2021^38^ (US)	‘You see that *they come from the health service to do some tests in the nursing home* or whatever and […] obviously, it’s *nice to see how they dress, undress, all new, they take it off,* they don’t touch anything. It’s *a little sad, in that sense, that some have so much and others so little*.’ (Nurse) (Romeu-Labaven, 2022, Spain) [*italics added by author*]
7	Individual health worker factors affecting PPE use	Donning PPE takes time and affects the ability to carry out clinical procedures	Goodarzi, 2022^30^ (Iran); Hoernke, 2021^37^ (UK); Sivaraman, 2022^27^ (India);	‘At times doctors are faced with the dilemma regarding ‘patient care first or personal safety first?’ *Donning of the PPE takes time. So, if a patient requires emergency care, there would be no time to put the PPE on*.’ (Sivaraman, 2022, India) [*italics added by author*] ‘It’s strangling, and *it’s lowering the quality of work*, particularly for us. *We feel exhausted during resuscitation.* It’s not at all comparable to the resuscitation in non-COVID patients and comfort of the clothes there … Using this equipment in resuscitation is tough. You may not believe that 5 minutes of resuscitation with this cover equals one hour of normal resuscitation.’ (Nurse) (Goodarzi, 2022, Iran) [*italics added by author*]
		PPE design and fit influences risk of contamination	Fan, 2020^26^ (China); Sharma, 2022^28^ (India)	‘When *unzipping, the front of the coverall often curled inward, making contact with the health workers’ scrubs and neck. Taking off the coverall requires a certain amount of force, which creates a risk of splash pollution.* After removing the outer gloves, the wrist skin was occasionally exposed, increasing the risk of infection’ … *‘PPE design does not enable easy distinction between the contaminated and clean sides of the items.* In addition, some areas were not well covered by PPE, for instance, *gowns were often too large and left parts of the neck exposed, even when sized appropriately. Participants’ backs occasionally became exposed during work.*’ (Fan 2020, China) [*italics added by author*] ‘… There are some PPEs, which come with a zip closure, and their quality is so bad that it rips apart when I wear them. Also, *we get different size of head caps and it is very loose which exposes us and hinders our vision while working*.’ (Sharma 2022, India) [*italics added by author*]
		Questions about the effectiveness of PPE	Fan, 2020^26^ (China); Sivaraman, 2021^27^ (India); Broom, 2022^25^ (Australia)	‘The impermeability and tightness of the covering, including connections between various parts of PPE, is a common concern. Some HCP described that they found it *difficult to assess the impermeability ratings* and described the body covering as a similar impervious material but without a logo or any hints. Three participants … *also wanted to obtain valid evidence of the duration of effectiveness of the PPE and the optimal frequency of replacement.*’ (Fan, 2020, China) [*italics added by author*]
8	Health system factors that affected PPE use	Availability and supply of PPE	Romeu-Labaven, 2022^34^ (Spain); Hoernke, 2021^37^ (UK); Setiawan, 2021^29^ (Indonesia)	‘I think the one thing that’s probably been *the biggest challenge has been sourcing PPE* … That was probably the *single biggest anxiety-inducing thing for staff on the ground.* … but there was *always this sense that we don’t know where next week’s is coming from* …’ (Hoernke, 2021, UK) [*italics added by author*]
		Gap between guidelines and protocols and implementation of PPE	Romeu-Labaven, 2022^34^ (Spain); Hoernke, 2021^37^ (UK); Fan, 2020^26^ (China); Broom, 2022^25^ (Australia)	‘*Because they can’t say 50 protocols* […] *perfectly describing what to do with PPE, and then not have PPE.* […] *I try to make people respect the protocol as much as possible, I’ll make use of what I have, right*? And I’ll adapt according to the general conditions or common sense in many things. But if you’re telling me to put on, to throw away the FFP2 every time or the FFP3. […] Because *protocol tells you that you have to throw it away every time you go in, but if you don’t have* [*enough*], *you can’t throw it away every time.*’ (Nursing supervisor) (Romeu-Labaven, 2022, Spain) [italics added by author]
9	Facility-level factors that affected PPE use	Institutional support and role modelling helped PPE use	Sivaraman, 2022^27^ (India)	‘The timely, regular release of updated guidelines by the institute that were aligned with the global scenario encouraged the HCP to abide by the PPE usage … In addition, regular “supervision” by senior doctors promoted PPE use. A junior resident told, “the *faculty were doing it and I did see them.*” *“Role modelling”* [*my seniors do; hence I should do*] worked here.’ (Sivaraman, 2022, India) [*italics added by author*]
		Layout of health facility affected PPE use	Fan, 2020^26^ (China); Sharma, 2022^28^ (India)	‘Participants also discussed how *the space layout influenced their ability to follow PPE protocols.* It seems that the *areas considered clean and contaminated varied across different HCP.* In fact, the rules pointed that red zone was within the wards and outside corridors were considered clean, but some individuals did not follow the rules strictly.’ (Fan, 2020, China) [*italics added by author*] ‘… Yes we have, but it is not enclosed. It is in an open area and I think there is no privacy due to which I do have a problem changing there.’ (Sharma, 2022, India)
10	Health and care workers responded creatively to the lack of masks and other PPE	Reusing, rationing and improvising with PPE or mask	Romeu-Labayen, 2022^34^ (Spain); Sivaraman, 2022^27^ (India)	‘We’ve suffered a lot over equipment. […] *Looking for tutorials on how to make gowns out of garbage bags.* The cleaning woman giving us large garbage bags. A midwife who knows a lot about sewing, making tutorials on how to do it. One [*nurse*] *who has a neighbour whose husband works in China giving her masks* … *My mother making cloth hats for* all my co-workers’ … I would sometimes go out sweaty, with my whole mask wet, dripping, and *wanting to change my mask and they* [*the supervisor*] *would say, “No! It has to last for a week*!”.’ (Romeu-Labayen, 2022, Spain) [*italics added by author*] ‘… I will *put my N95 mask in a paper cover. And I put it aside without touching, to do anything else. And once the 1 week is over, I’ll use it* …’ (Sivaraman, 2022, India) [*italics added by author*]
		Procurement and quality control of masks	Venesoja, 2021^31^ (Finland); Martinelli, 2020 (20 Euro countries, China, S Korea); Broom, 2022^25^ (Australia); Sharma, 2022^28^ (India)	‘Very much *varying ‘guts’ about quality, some of them feel very high quality and safe* in the face while *some are like any kind of rag* piece in the face.’ (Venesoja, 2021, Finland) [*italics added by author*] ‘… I have experienced that PPE does not sustain and gets torn if I wear it for more than 3 hours.’ (Sharma 2022, India)
11	Solidarity in not physically distancing	Peer and social pressure to comply (or not) with physical distancing	Berry, 2022 (UK); Farrell, 2021 (Ireland); Eraso, 2021 (UK); Fauk, 2022^42^ (Indonesia)	‘I have often seen *there are people who do not want to wear masks or keep social distancing in social events* they attend, and even influence their friends or family members not to comply with COVID-19 guidelines. Some said to their neighbours ‘it is not necessary to wear masks. *We know each other, so why we should wear masks all the time and keep distance or sit far from each other* …’ (Fauk, 2022, Indonesia) [*italics added by author*]
		Difficulty complying with physical distancing among friends and family	Berry, 2022 (UK); Fauk, 2022^42^ (Indonesia); Keller, 2022^43^ (US)	‘We’re all friends in the hospital so it’s hard to [*physically distance*]. I think *it’s a sense of camaraderie also to not social*[*ly*] *distance*. People say things like “Let’s do a contraband hug or illicit hug,” and that’s a sign of endearment’… *we tend to be more lax [with physical distancing] than strict just because … this is the only contact we have with the outside world* a lot of times.’ (Keller, 2022, US) [*italics added by author*]
12	Physical distancing is a barrier to relational care	Physical distancing affects relationships with patients	Kane, 2022^41^ (Belgium, France, Italy, Luxembourg); Stulz, 2022^40^ (Australia); Ferrari, 2021^33^ (Italy)	‘I don’t try to stay a hard one point five metres away from the women I’m caring for all the time. Because I can’t help them breastfeed that way, and *I can’t effectively reassure somebody from one point five metres away* if it looks like you’re keeping your distance. *That’s not actually part of how relational care works*.’ (Stulz, 2022, Australia) [*italics added by author*]
13	Difficult to challenge those higher up the hierarchy	Workplace hierarchy influences social distancing	Keller, 2022^43^ (US)	‘It was *difficult for HCWs to ask those higher in the hierarchy, or in a different hierarchy* [*e.g., resident physicians versus nurses*] *to physically distance*. Hierarchy even determined where HCWs sat … However, leaders promoted physical distancing … *higher up physicians, those guys are pretty good at being responsible and … calling out when we need to remain physically distanced.*’ (Keller, 2022, US) [*italics added by author*]
14	Physical infrastructure prevents physical distancing	Health facility infrastructure prevents physical distancing	Keller, 2022^43^ (US); Berry, 2022 (UK)	‘So, *we were told we should socially distance but there weren’t enough chairs in the offices*. We were *told to socially distance, but the computers were not spaced such that we can do that*. And so suddenly, if there were 7 computers in the room, to socially distance we could only use three.’ (Keller, 2022, US) [*italics added by author*]
15	Service delivery presents barriers to physical distancing	Clinical workflows require health workers to gather in proximity	(Keller, 2022^43^ US)	‘When you have *beginning-of-the-shift huddles that makes it a little harder to physically distance* yourself when you have now *12 or 13 people between the day shift and night shift together*.’ (Keller, 2022, US) [*italics added by author*]

Note: Please see the full reference list of the article Arikpo DI, Oku AO, Onyema OA, et al. Health and care workers’ perceptions of PPE and physical distancing for COVID-19: A qualitative evidence synthesis. J Public Health Africa. 2025;16(2), a621. https://doi.org/10.4102/jphia.v16i2.621, for more information.

PPE, personal protective equipment; COVID, coronavirus; ICU, intensive care unit; UK, United Kingdom; US, United States; PAPRs, powered air-purifying respirators; HCP, healthcare personnel.

**TABLE 4:** Summary of qualitative findings table: Personal protective equipment use and physical distancing in healthcare settings.

S/N	Summary of review finding (analytical theme)	Studies contributing to this review finding	CERQual assessment	Explanation of CERQual assessment
1	Health and care workers value the use of masks and PPE in patient care	Sivaraman, 2022^27^ (India); Ribeiro, 2021^32^ (Portugal)	Moderate confidence	No to very minor concerns regarding methodological limitations and coherence, minor concerns regarding adequacy and moderate concerns regarding relevance (limited geographical spread)
2	Health and care workers were anxious about being put at risk	Broom, 2022^25^ (Australia); Hoernke, 2021^37^ (UK), Sivaraman, 2022^27^ (India)	Moderate confidence	No to very minor concerns regarding methodological limitations and coherence, minor concerns regarding adequacy and moderate concerns regarding relevance (limited geographical spread)
3	Masks and other PPE cause physical discomfort to health and care workers	Setiawan, 2021^29^ (Indonesia); Broom, 2022^25^ (Australia); Sivaraman, 2022^27^ (India); Sharma, 2022^28^ (India); Hoernke, 2021^37^ (UK); Fan, 2020^26^ (China); Koken, 2022^35^ (Turkey); Venesoja, 2021^31^ (Finland); Markkanen, 2021^38^ (US); Ribeiro, 2021^32^ (Portugal)	High confidence	No to very minor concerns regarding methodological limitations, coherence and adequacy, minor concerns regarding adequacy and minor concerns regarding relevance (moderate geographical spread)
4	Masks and other PPE challenge effective communication	Hoernke, 2021^37^ (UK); Hayirli, 2021^36^ (US); Koken, 2022^35^ (Turkey); Ferrari, 2021^33^ (Italy); Setiawan, 2021^29^ (Indonesia); Sivaraman, 2022^27^ (India); Clay, 2022^39^ (UK); Markkanen, 2021^38^ (US); Ribeiro, 2021^32^ (Portugal)	High confidence	No to very minor concerns regarding methodological limitations, coherence and adequacy, minor concerns regarding adequacy and minor concerns regarding relevance (moderate geographical spread)
5	Familiarity with masks and other PPE helped health workers adapt to COVID-19	Sivaraman, 2022^27^ (India); Ribeiro, 2021^32^ (Portugal)	Moderate confidence	No to very minor concerns regarding methodological limitations and coherence, minor concerns regarding adequacy and moderate concerns regarding relevance (limited geographical spread)
6	Health and care workers in care homes experienced unequal access to PPE	Romeu-Labaven, 2022^34^ (Spain), Markkanen, 2021^38^ (US)	Moderate confidence	No to very minor concerns regarding methodological limitations and coherence, minor concerns regarding adequacy and moderate concerns regarding relevance (all studies conducted in high-income settings)
7	Individual health and care worker factors affecting PPE use	Goodarzi, 2022^30^ (Iran); Hayirli, 2021^36^; Hoernke, 2021^37^ (UK); Sivaraman, 2022^27^ (india); Fan, 2020^26^ (China); Broom, 2022^25^ (Australia)	High confidence	No to very minor concerns regarding methodological limitations, coherence and adequacy, minor concerns regarding adequacy and minor concerns regarding relevance (moderate geographical spread)
8	Health system factors that affected PPE use	Romeu-Labaven, 2022^34^ (Spain); Hoernke, 2021^37^ (UK); Setiawan, 2022^29^ (Indonesia), Fan, 2020^26^ (China); Broom, 2022^25^ (Australia); Sharma, 2022^28^ (India)	Moderate confidence	No to very minor concerns regarding methodological limitations, coherence and adequacy, minor concerns regarding adequacy and minor concerns regarding relevance (moderate geographical spread)
9	Facility-level factors that affected PPE use	Sivaraman, 2022^27^ (India), Sharma, 2022^28^ (India); Fan, 2020^26^ (China)	Low confidence	No to very minor concerns regarding coherence, minor concerns regarding methodological limitations, and adequacy, moderate concerns regarding relevance (limited geographical spread)
10	Health and care workers responded creatively to the lack of masks or other PPE	Romeu-Labayen, 2022^34^ (Spain); Sivaraman, 2022^27^ (India), Sharma, 2022^28^ (India), Venesoja, 2021^31^ (Finland)	Moderate confidence	No to very minor concerns regarding coherence, minor concerns regarding methodological limitations, relevance and adequacy
11	Solidarity in not physically distancing	Fauk, 2022^42^ (Indonesia); Keller, 2022^43^ (US)	High confidence	No to very minor concerns regarding methodological limitation, coherence and adequacy, minor concerns regarding relevance
12	Physical distancing is a barrier to relational care	Kane, 2022^41^ (Belgium, France, Italy, Luxembourg); Stulz, 2022^40^ (Australia); Ferrari, 2021^33^ (Italy)	Moderate confidence	No to very minor concerns regarding methodological limitations and coherence, minor concerns regarding adequacy and moderate concerns regarding relevance
13	Difficult to challenge those higher up the hierarchy	Keller, 2022^43^ (US)	Low confidence	No to very minor concerns regarding methodological limitations and coherence, moderate concerns regarding relevance and adequacy
14	Physical infrastructure prevents physical distancing	Keller, 2022^43^ (US)	Moderate confidence	No to very minor concerns regarding methodological limitations and coherence, minor concerns regarding adequacy and moderate concerns regarding relevance
15	Service delivery presents barriers to distancing	Keller, 2022^43^ (US)	Low confidence	No to very minor concerns regarding methodological limitations and coherence, moderate concerns regarding relevance and adequacy

Note: Please see the full reference list of the article Arikpo DI, Oku AO, Onyema OA, et al. Health and care workers’ perceptions of PPE and physical distancing for COVID-19: A qualitative evidence synthesis. J Public Health Africa. 2025;16(2), a621. https://doi.org/10.4102/jphia.v16i2.621, for more information.

PPE, personal protective equipment; UK, United Kingdom; US, United States.

#### Personal protective equipment including mask use in healthcare settings

*Finding 1: Health and care workers value the use of masks and other PPE in patient care (moderate confidence)*: Health and care workers in India (LMIC) and Portugal (HIC),^27,32^ valued the use of masks and other PPE in patient care. They reported experiencing ‘peace of mind’ and reduced anxiety and fear when they put on their PPE. A nursing officer explained that the feeling was because the PPE covered the ‘entire body’.^27^ In addition, the health and care workers reported that *masks and other PPE created a safety climate to deliver optimal patient care.* Psychotherapists felt safe, especially when they had to provide face-to-face therapy.^32^ By putting on PPE, health and care workers felt they faced less risk of contracting the virus.

*Finding 2: Health and care workers were anxious about being put at risk* (*moderate confidence*): Despite the value placed on PPE by health and care workers in one LMIC and two HICs, they experienced *anxiety and insecurity about the level of protection provided by PPE*.^25,27,37^ An intensive care unit nurse expressed concern that the type and quality of PPE gear provided to health and care workers varied across settings with equal risk. Early in the pandemic, the media showed some health and care workers in ‘full PPE’, including hazmat suits and hats, even though these were not required. A consultant doctor expressed a similar dissatisfaction with the recommended PPE.^25,37^ There was rampant comparison of PPE across healthcare settings; therefore, health and care workers who had access to other forms of PPE – gowns, N95 masks and goggles – felt unprotected given this media attention. Consequently, health and care workers in settings without hazmat suits had heightened perceptions of susceptibility and risk and felt less prioritised compared to health and care workers from other settings. Moreover, they described that they were *most anxious about endangering their families*.^25,35^

*Finding 3: Masks and other PPE cause physical discomfort to health and care workers (high confidence*): Experiencing physical discomfort while wearing masks and other PPE was reported in 10 studies across LMICs, UMICs and HICs.^25,26,27,28,29,31,32,35,37,38^ This experience appeared common across different categories of health and care workers. The discomfort was variously described in the studies to include fatigue, sweating, headaches, overheating, a feeling of suffocation, perceived burning sensation in the lungs, dehydration, tight bands, pressure on the ears, abrading behind the ear, skin irritation, foggy eyeglasses and exhaustion. Of note, health and care workers reported perceived difficulties with breathing when wearing respirators. Ill-fitting PPE (including masks and gowns) were also reported to contribute to the discomfort experienced by health and care workers; PPE was described as either too tight, oversized or undersized. Health and care workers who wore oversized or undersized PPE felt that they were not optimally protected because the PPE snapped open at intervals and exposed parts of their bodies. Health and care workers explained how they suppressed the desire to take bathroom breaks or meal breaks when wearing PPE because stepping out of the wards implied disposing of the already short-supplied PPE.^29^

*Finding 4: Masks and other PPE challenge effective communication* (*high confidence*): In nine studies,^27,29,32,33,35,36,37,38,39^ health and care workers described how PPE compromised effective communication. Health and care workers in HICs and middle-income countries (MICs) reported they could not hear each other while wearing PPE and noticed they had to speak repetitively and loudly. The inability to recognise or read colleagues’ facial expressions limited non-verbal communication among healthcare teams affecting teamwork. They also described how masks and other PPE affected communication with recipients of care who struggled to hear or understand the health and care workers, which often resulted in misunderstandings and arguments. Health and care workers also experienced that PPE use limited their ability to establish relationships with recipients of care because the recipients could not decipher the facial expressions of their healthcare provider. They described how children, in particular, were scared when approached by health and care workers wearing PPE. Of note, health and care workers revealed how they removed their masks to overcome the communication limitations when caring for children, especially when the procedures were painful. Some health and care workers also outlined how they learnt *how to communicate effectively with patients while wearing a mask* by speaking slowly, eliciting feedback from care recipients and educating care recipients on the importance of PPE during the provision of care. Furthermore, masks compromised the ability to build human connections and engendered distrust when interacting with health and care workers because of the inability of recipients of care to read their facial expressions.

*Finding 5: Familiarity with masks and other PPE helped health and care workers adapt to COVID-19* (*moderate confidence*): Health and care workers recounted that it was easier to adapt to masks and other PPE during the pandemic because they were *already familiar with PPE pre-pandemic*.^27,32^ For example, otorhinolaryngologists had hitherto used masks during tracheostomy. Some health and care workers were already familiar with PPE use, for example, some health and care workers reported using PPE found in HIV kits, which ‘containing safety goggles, gloves, double mask, surgical gown and leg cover’ in surgical procedures involving patients with blood-borne infections, provided familiarity in the context of COVID-19. Therapists also described that they experienced progressive adaptation over time with mask use despite the difficulties of providing therapy in these circumstances.

*Finding 6: Health and care workers in care homes experienced unequal access to PPE* (*moderate confidence*): Health and care workers in a nursing home in a study conducted in Spain and those delivering home healthcare in the US described that they felt less prioritised by the government and health authorities even though they faced an equivalent risk of infection with SARS-CoV-2.^34,38^ They recounted how health and care workers from hospital settings had access to a broader range of PPE, but they did not. They had received training on COVID-19 protocols but no PPE.

*Finding 7: Individual health and care worker factors affecting PPE use* (*high confidence*): In seven studies,^25,26,27,28,30,36,37^ health and care workers in Australia, China, India, Iran and the UK experienced that *putting on PPE takes time and affects the ability to carry out clinical procedures*. They described having limited time to put on PPE before providing emergency care and, as such, faced the dilemma of either prioritising patient care or personal safety. Some health and care workers also experienced that procedures such as administering cardiopulmonary resuscitation (CPR) were more difficult and exhausting and took more time when they wore PPE compared to when they did not put PPE on; such that they worried that it affected resuscitation efficiency and outcomes. Health and care workers also reported that they *questioned the effectiveness of PPE* and the extent of protection provided by different types of masks. The questions included the duration of the effectiveness of PPE, the effectiveness of level two masks compared to level three masks as defined by the America Society for Testing and Materials (ASTM),^45^ and the frequency of replacement. Health and care workers in China felt that *the design and fit of PPE influenced the risk of infection*.^26^ Inappropriately fitted PPE, for example, oversized PPE, was perceived to increase the risk of contamination because it dragged across surfaces during healthcare and exposed parts of the face or body. In contrast, undersized PPE created discomfort because of poor fit.

*Finding 8: Health system factors that affected PPE use* (*moderate confidence*): Some health system factors were perceived to influence PPE use at the onset of the pandemic. Two sub-themes describe this finding from six studies conducted in HICs and MICs.^25,26,28,29,34,37^ These factors included the *unavailability and short supply of PPE,* which was reported to affect PPE use by health and care workers and clients. For example, the acute scarcity and rationing of PPE resulted in poor adherence to the guidelines and the reported rationing and reuse of PPE. Healthcare institutions were also confronted by the additional challenge of providing PPE for their workers. Some healthcare institutions took it upon themselves to source PPE for their staff because they were running out rather than waiting for rations from health authorities. The scarcity of PPE caused healthcare institutions to accept donations of PPE despite being unsure if they met acceptable standards and offered adequate protection. This situation of making do with what was available highlighted *the gap between prescribed guidelines and the implementation of PPE.* Especially at the onset of the COVID-19 pandemic where governments and health bodies prescribed strict rules but did not supply the PPE to health and care workers to enable implementation of the guidance. Health facilities were also unable to provide PPE for family members who went in to visit loved ones receiving end-of-life care.

*Finding 9: Facility-level factors that affected PPE use* (*low confidence*): Three studies conducted in China (UMIC) and India (LMIC) highlighted factors at the facility-level that affected PPE use.^26,27,28^ *Institutional support* in the form of regular and timely communication of updated guidelines on PPE use and role modelling (proper use of the PPE by senior faculty members) was perceived to motivate staff to comply with the guidelines. Furthermore, the *layout of health facilities affected PPE use* as experienced by the health and care workers. This was largely because some facilities lacked clarity on clean and contaminated (red) zones, causing health and care workers to put on or take off their PPE in inappropriate places. Female health and care workers also expressed reluctance in using the common changing room for putting on and taking off PPE.^28^ This feeling was because of the lack of privacy because males and females used the same common changing room.

*Finding 10: Health and care workers responded creatively to the lack of masks or other PPE* (*moderate confidence*): In three studies conducted in India (LMIC)^27,28^ and Spain (HIC),^34^ health and care workers reported that they resorted to *reusing, rationing and improvising with PPE or masks* when PPE was unavailable. Although not the best practice, health and care workers reported reusing wet and sweat-filled masks during shifts. Some health and care workers also described how they had to label their masks with the date of use and allowed a fallow period of 1 week before reusing them; others disinfected their PPE with bleach and an ozone machine. There were also cases of improvising by making gowns out of trash bags and plastics, adapting diving goggles for use as PPE and having their parents sew cloth hats. Health and care workers in two HICs and one LMIC also experienced that the quality of PPE supplied to them varied.^25,28,31^ This experience generated concerns about mask procurement and quality control processes. Some masks and other PPE were of high quality, and health and care workers were more comfortable using them; others were of lower quality and tore between uses, exposing the workers to the risk of infection.

#### Physical distancing interventions

*Finding 11: Solidarity in not physically distancing* (*high confidence*): Health and care workers in one HIC and another LMIC experienced *difficulty complying with physical distancing among colleagues, friends and family*.^42,43^ This was because of *peer and social pressure* and the ‘sense of camaraderie’ in not observing physical distancing while interacting with family or acquaintances. Health and care workers who had worked closely during shifts or on a patient found it contradictory to enforce physical distancing among themselves when they were out of the ward. The actions of others – neighbours, friends and colleagues were also reported to influence adherence with physical distancing guidelines. Concerns about how ethical it was to insist that a person stays away during an interaction were also expressed by participants.

*Finding 12: Physical distancing is a barrier to relational care* (*moderate confidence*): Health and care workers in three studies from HICs experienced physical distancing as a barrier to relational care.^33,40,41^ Midwives described how they found it challenging to provide breastfeeding support to new mothers and struggled to empathise with women during childbirth because they could not touch them. Nurses reiterated that some recipients of care, for example, the elderly respond well to touching and other physical gestures, which were limited by physical distancing. For psychologists, physical distancing guidelines did not adequately support the rehabilitation and resocialisation of patients. The use of physical barriers such as plastic screens between health and care workers and clients or patients was also perceived to introduce a disconnect between health and care workers and patients.

*Finding 13: Difficult to challenge those higher up the hierarchy* (*low confidence*): In one HIC study,^43^ health and care workers willing to observe physical distancing recounted that they were limited by power dynamics in the workplace. Younger colleagues found it challenging to request seniors to maintain physical distance when non-adherence was observed.

*Finding 14: Physical infrastructure prevents physical distancing* (*moderate confidence*): *Physical infrastructure prevented physical distancing* in a healthcare setting. This finding was reported by one study conducted in a HIC.^43^ Participants expressed that the infrastructure in healthcare facilities was not readily amenable to physical distancing or spatial separation requirements at the onset of the pandemic; therefore, complying with physical distancing guidelines was not feasible. For instance, there was no spatial separation of 1 metre or 2 metres between computers and workstations in healthcare settings to facilitate adherence to physical distancing. Skipping computers to maintain physical distancing limited work efficiency by reducing the number of computers available for use by health and care workers.

*Finding 15: Service delivery presents barriers to physical distancing* (*low confidence*): In one study conducted in a HIC,^43^ health and care workers reported that clinical workflows made it difficult to adhere to physical distancing. *Clinical workflows require health and care workers to gather in proximity* to receive guidance for consecutive shifts, especially at the beginning of shifts when health and care workers converge for note-taking, briefing and handing over to the next team. Health and care workers also experienced that physical distancing was not feasible during patient rounds and when there was the need to share patient information that was considered confidential. These service delivery activities precluded staying at a distance from one another.

## Discussion

Overall, health and care workers valued the use of PPE during patient care. The sense of value was heightened by perceived susceptibility to infection with COVID-19, the need to deliver optimal patient care and the desire to protect family members. These factors contributed to the acceptability of PPE and the willingness of health and care workers to use them. Health and care workers also value the ability to communicate among themselves and with their patients, and provide relational patient care. The use of PPE compromised that ability in most cases, with some health and care workers breaching IPC guidelines in order to foster communication (e.g., when caring for patients with hearing impairment). Health and care workers struggled to navigate these communication difficulties, which were reported across the geographical settings. Furthermore, health and care workers in various settings reported different forms of discomfort and difficulties when they used PPE and issues about the fit and quality of PPE. There is the need for design and supply of PPE of optimum quality that enhances comfort, supports effective communication and is easy to wear.

The need to socialise among health and care workers, and provide relational patient care were limitations to the uptake of physical distancing interventions by health and care workers. This finding suggests that physical distancing was not readily acceptable to health and care workers largely because of its effects on relationships. At the health system level, service delivery, clinical workflows, the absence of visual cues for spatial separation, a limited number of workstations and physical infrastructure hindered adherence to physical distancing guidelines. It was not feasible to observe physical distancing during clinical work and service delivery. This QES also identified equity issues in the implementation of PPE and physical distancing. Health and care workers in care homes experienced inequity in access to PPE and felt less prioritised during the pandemic. Workplace hierarchy limited the ability to advocate to senior colleagues to observe physical distancing.

### Implications for practice

Our review findings suggest that health system and facility-level factors, including PPE availability and supply, PPE quality control and procurement, health facility layout, inadequate spatial separation and the gap between IPC guidelines and their implementation are principal factors affecting adherence with PPE and physical distancing measures in the context of COVID-19. It is, therefore, critical to explore avenues for improving the experiences of health and care workers and addressing the barriers to adherence. Such measures could include innovative PPE design to reduce the physical discomfort and communication difficulties associated with wearing PPE, designation of areas for putting on and taking of PPE, ensuring availability of high quality PPE through adequate quality control and improvement in procurement processes, the use of visual cues for space awareness and improving health facility infrastructure to support physical distancing. Risk assessment for health and care workers in care homes may be required to reduce inequities in PPE access. Although issues around the fit of PPE may appear a complex problem given the diversity of the physical features of health and care workers, healthcare facilities managers should consider the characteristics of their workforce during PPE procurement.

### Overall completeness of the evidence and implications for research

The sampled studies traverse different socio-economic settings and countries, as this was one of the criteria for sampling studies in the review. However, only a few study reports were identified from low-income countries (LICs) and MICs. The majority of the studies on PPE and masks did not specify the type of PPE or mask respondents referred to; therefore, we are unable to narrow the findings down to a specific type of PPE. The scope of physical distancing in this review was limited to maintaining a distance of 1 m or 2 m between individuals; we did not include studies that reported on the perspectives of the ‘lockdown’.

This review identified the following research gaps. Only two studies conducted in HICs contributed evidence for care homes. The evidence on physical distancing was also provided by studies conducted in HICs. This QES also sought to summarise evidence on the perceptions of physical distancing among inpatients and engineering controls, but none of the included studies reported these interventions. Finally, studies in healthcare settings did not assess the perspective or experiences of healthcare administrators with organising workflows, procurement and infrastructure for healthcare delivery during the pandemic. Although the QES included studies conducted in LMICs, which provides some basis for comparison, we did not identify any studies set in Africa. Primary research addressing these gaps is needed to further broaden our understanding of adherence with IPC measures in the context of COVID-19.

### Limitations of the study

This rapid review was conducted in response to the update for IPC guidelines for COVID-19. In keeping up with the rapid review methodology, we searched only one database – Ovid MEDLINE. We also limited our search to studies published in English language published within a specific period and analysed only a representative sample of studies. These steps may have limited the range of relevant studies we identified, assessed for eligibility and included in this QES. Notwithstanding these limitations, the review team maintained methodological rigour in conducting the review using standard Cochrane methods, which reduced the bias that may be associated with studies of this nature. Majority of studies sampled in this QES were from UMICs and HICs. The three studies from two LMICs contributed moderately to the review findings. We did not identify any studies set in Africa. There was also insufficient evidence from care homes.

## Conclusion

This QES reports 15 findings of low to high confidence. Our review findings suggest that adherence to PPE use and physical distancing guidelines were influenced by individual, social and institutional factors. The gap between guidelines and their implementation stood out as an important health system barrier to PPE use across regions. The uptake of physical distancing interventions was primarily hindered by the lack of physical infrastructure to aid space awareness and spatial separation. This QES outlines these factors for consideration by public health authorities given their implications for future emergency preparedness and response. It is critical to explore avenues for improving the experiences of health and care workers when using PPE and remove institutional barriers that hinder adherence to public health guidelines.

## Appendix 1

### Medline search strategy

Date of search: 05 September 2022

1. Air Filters/ or Air Ionization/ or Ventilation/
2. (HVAC or HEPA or MERV or CADR or HRV or ERV or &quot;Heating ventilation and air conditioning&quot; or &quot;High Efficiency Particulate Air&quot; or &quot;High Efficiency Particulate Arrestance&quot; or &quot;Minimum Efficiency Reporting Value&quot; or &quot;Clean Air Delivery Rate&quot; or &quot;Heat Recovery Ventilator&quot; or &quot;Energy Recovery Ventilator&quot;).ti,ab,kf.
3. (ventilation adj2 (unit* or system* or technolog* or vents or equipment or device*)).ti,ab,kf.
4. (air adj2 (cleaner* or cleaning or filter* or filtering or filtration or purifier* or purifying or purification or ultraviolet or ultra violet or UV or UVGI or UVC or actinic or ioni?er* or ioni?ing or ioni?ation or ozone or ozoni?er* or ozoni?ation)).ti,ab,kf.
5. (ion generator* or electrostatic precipitator*).mp.
6. or/1-5
7. air pollutants/ or air pollutants, occupational/
8. air pollution/ or air pollution, indoor/
9. Air/ or Air Microbiology/
10. Aerosols/ or Inhalation Exposure/
11. or/7-10
12. filtration/ or Ultraviolet Rays/ or Ultraviolet Therapy/
13. 11 and 12
14. 6 or 13
15. patient isolators/ or protective devices/ or masks/ or personal protective equipment/ or exp protective clothing/ or exp respiratory protective devices/
16. ((protective adj2 (clothing or equipment or device?)) or PPE or glove? or gown?).ti,ab,kf.
17. (mask? or facemask* or &quot;face mask*&quot; or respirator? or N95 or N99 or FFP2 or FFP3 or &quot;Filtering face piece?&quot; or &quot;Filtering facepiece?&quot; or faceshield? or &quot;face shield?&quot; or goggles).ti,ab,kf.
18. or/15-17
19. ((engineering or environmental) adj2 control?).ti,ab,kf.
20. physical distancing/
21. ((physical* or social* or spa?ial) adj2 (barrier? or distanc* or separation or proximity)).ti,ab,kf.
22. (distancing adj2 (intervention? or measure?)).ti,ab,kf.
23. or/19-22
24. 14 or 18 or 23
25. limit 24 to covid-19
26. Health Knowledge, Attitudes, Practice/ or health behavior/ or health risk behaviors/ or exp

Guideline Adherence/

27 (&quot;adhere to&quot; or adherence or attitude* or barriers or challeng* or compliance or comply*or facilitat* or influenc* or knowledge or perception* or practice*).ti,ab,kf.
28 26 or 27
29 25 and 28
30 exp animals/ not humans.sh.
31 29 not 30
32 (comment or editorial or newspaper article).pt.
33 31 not 32
34 limit 33 to english language
35 limit 34 to &quot;qualitative (maximizes specificity)&quot;
